# Systemic Air Embolism Associated with Pleural Pigtail Chest Tube Insertion

**DOI:** 10.1155/2016/4053748

**Published:** 2016-08-17

**Authors:** Emad Alkhankan, Ahmad Nusair, Rida Mazagri, Mohammed Al-Ourani

**Affiliations:** ^1^Internal Medicine, Marshall University, Huntington, WV 25701, USA; ^2^Infectious Disease, Marshall University, Huntington, WV 25701, USA; ^3^Neurosurgery, Marshall University, Huntington, WV 25701, USA; ^4^Pulmonary, Marshall University, Huntington, WV 25701, USA

## Abstract

Pleural pigtail catheter placement is associated with many complications including pneumothorax, hemorrhage, and chest pain. Air embolism is a known but rare complication of pleural pigtail catheter insertion and has a high risk of occurrence with positive pressure ventilation. In this case report, we present a 50-year-old male with bilateral pneumonia who developed a pneumothorax while on mechanical ventilation with continuous positive airway pressure mode. During the placement of the pleural pigtail catheter to correct the pneumothorax, the patient developed a sudden left sided body weakness and became unresponsive. An air embolism was identified in the right main cerebral artery, which was fatal.

## 1. Introduction

Pleural effusion and pneumothorax are well-established indications for chest tube placement. Two widely known corrective procedures are using a pigtail catheter drainage or performing a chest tube thoracostomy. The pigtail catheter drainage is more widely used as it is easier to perform in emergent situations and is a much lesser invasive procedure [1–3]. Cerebral air embolism is a rare complication that can be induced by pulmonary barotrauma, trauma of the chest or head, and iatrogenic causes such as invasive procedures or surgery [4]. This risk is further increased if the patient is on positive pressure ventilation (PPV) as the pressure in the airway is much higher. Despite its rarity, there are many reports written on cerebral air embolism. However, reports on the occurrence of cerebral air embolism while the patient is on positive pressure ventilation (PPV) remain scarce.

## 2. Case Report

This is a case of a 50-year-old male with no significant past medical history, who initially presented with shortness of breath and hypoxia. He was transferred to the intensive care unit (ICU) and was treated for bilateral pneumonia that required prolonged mechanical ventilation via a tracheostomy. He further developed necrotizing pneumonia and subsequently multiorgan failure that led to hospitalization for 6 weeks. He was weaned from mechanical ventilation to the point he was tolerating CPAP mode. However, he started requiring higher fraction of inspired oxygen (FiO_2_). A chest X-ray (CXR) showed a small pneumothorax and was shown to increase in size in a repeat CXR the following day. This led to the decision to place a pleural pigtail chest tube to relieve the pneumothorax.

**Figure 1:**
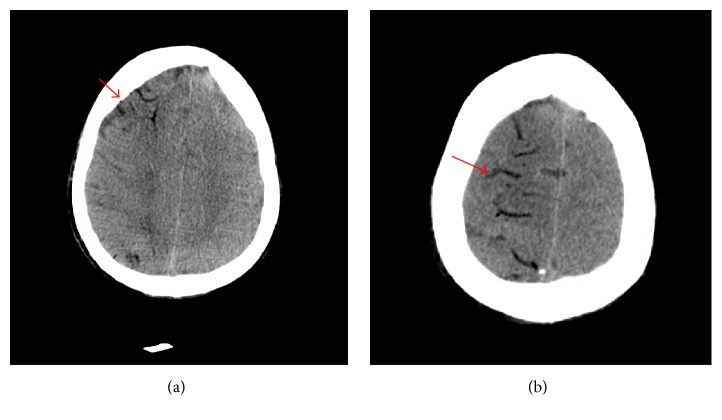
Computed tomography (CT) imaging of the brain showing air accumulation in right hemisphere with edema (arrows).

During the procedure, the patient suddenly became unresponsive and had an episode of apnea. However, his vital signs remained stable (BP 125/68 mmHg, P 84 BPM, T 37.0 C, RR 25 breaths/min, and SpO_2_ 95%). Additional labs included ABG (pH 7.37, PaCO_2_ 58 mm Hg, and PaO_2_ 67 mmHg), WBC 14,700/mm^3^, Hgb 8.4 g/dL, Plts 256 × 10^9^/L, Na 126 mmol/L, K 5.1 mmol/L, Cl 99 mmol/L, HCO_3_ 30.8 mmol/L, BUN 26 mmol/L, Cr 0.52 mmol/L, and Gluc 108 mmol/L. Another CXR did not show worsening of the existing pneumothorax and confirmed that the pleural pigtail catheter was in the appropriate position. However, an urgent computed tomography (CT) scan of the head showed low-density opacities that are consistent with air in a watershed pattern on the right hemisphere with edema (Figure 1).

The patient was returned on mechanical ventilation with assist control (AC) mode on high oxygen flow. He was placed in Trendelenburg position and was treated conservatively. Funduscopic examination showed no abnormality in the retina such as an air embolism. A follow-up computed tomography angiography (CTA) of the head and neck was done and showed that the air embolism had resolved. However, the patient's neurological status did not improve. A magnetic resonance imaging (MRI) of the brain confirmed large bilateral cerebral infarcts, which was greater on the right. Additionally, the MRI also showed infarcts developing within the pons and midbrain. Despite attempts to stabilize the patient, he expired two hours after insertion of the pleural pigtail catheter.

## 3. Discussion

Small-bore pigtail chest tubes (Seldinger type) potentially carry a high risk for complications such as hemothorax, pneumothorax, and pain. Being a procedure that involves insertion of a foreign body, there is also risk of injuring the visceral organs, which could lead to patient demise [5, 6].

Most systemic air embolism complications that have been reported were secondary to procedures such as percutaneous transthoracic needle biopsy, thoracentesis, chest trauma, and cardiopulmonary resuscitation [7]. However, cerebral air embolism as a complication of pigtail chest tube insertion for treatment of a pneumothorax is rare [2].

Many mechanisms of the occurrence of cerebral air embolism have been speculated. Air entering the circulation has to come from the atmosphere, airway tracts, or the air from the pneumothorax space. A few studies present that puncturing the lung parenchyma during the insertion of the chest tube can facilitate a gas bubble entry to the pulmonary venous system. The needle can directly puncture the pulmonary vein and expose it to the atmosphere, which has a pressure that greatly exceeds that of the pulmonary venous system [8–10]. In other cases, gas may enter the venous system from the nearby air-containing space when the needle penetrates both the air space and the vein. This can create a connection that facilitates the entry of a gas bubble into the venous circulation during the insertion of a chest tube. This can especially occur in patients who are requiring PPV, as PPV increases the patient's airway pressure [9–11].

In our case, one of these mechanisms may have occurred. However, since our patient was on positive mechanical ventilation, it was more likely that a gas bubble entered the venous system from a nearby air-containing space airway or pneumothorax space.

Air bubbles in the systemic arterial circulation can block any arterioles with a diameter of 30–60 *μ*m. However, the diagnosis of an air embolism is often difficult. Although the diagnosis can be made by the demonstration of air in the intravascular space, the air may also rapidly dissolve in the blood while awaiting diagnostic imaging [2, 12].

Air embolism is typically a retrospective clinical diagnosis. It is mostly based upon a high suspicion and the exclusion of other life-threatening processes. It is important to suspect cerebral air embolism in a patient who is having neurological symptoms during a pleural catheter procedure. A brain CT scan can confirm the diagnosis by showing air bubbles in the cerebral arteries. Also, a funduscopic examination may show the air bubbles in the vasculature of the eyes [10, 13].

If cerebral air embolism is suspected, the patient must start on high-flow oxygen therapy to increase tissue oxygenation, which helps in reducing the embolic volume by eliminating nitrogen [14]. When suspecting venous air embolization, the patient must be placed into the left lateral decubitus position with the head down or in Trendelenburg position in order to prevent air from entering the systemic arterial circulation by remaining in the superior aspect of the left ventricle and, away from the aorta [2, 15]. Giving hyperbaric oxygen therapy (HBOT) helps in reducing the volume of air emboli by increasing the solubility of oxygen in the blood and altering the permeability of blood-brain barrier, which decreases cerebral edema [9, 16]. In our case, the patient did not receive HBOT because he underwent mechanical ventilation after developing poor mental function and he also had an existing pneumothorax that was unresolved at that point.

## 4. Conclusion

Cerebral air embolism as complication of pigtail catheter is rare, but it is fatal. It should be suspected when the patient starts having neurological symptoms while undergoing chest tube insertion or other known chest procedures, especially while the patient is on positive pressure ventilation.
